# Activation of the HPV16 late promoter by viral E2 and cellular BRD4 and ZC3H4 proteins

**DOI:** 10.1128/jvi.00762-25

**Published:** 2025-08-13

**Authors:** Janis Renner, Karsten Boldt, Andreas Wieland, Adam Grundhoff, Thomas Guenther, Patrick Bluemke, Frank Stubenrauch, Thomas Iftner

**Affiliations:** 1 Institute for Medical Virology and Epidemiology of Viral Diseases, University Hospital Tuebingen, Eberhard Karls University Tuebingen 493697 , Tuebingen, Germany; 2 Institute for Ophthalmic Research, Eberhard Karls University Tuebingen, Tuebingen, Germany; 3 Department of Otolaryngology, Pelotonia Institute for Immuno-Oncology, The Ohio State University Comprehensive Cancer Center, The Ohio State University College of Medicine, The Ohio State University Wexner Medical Center, Columbus, Ohio, USA; 4 Leibniz Institute of Virology 28367, Hamburg, Germany; College of Agriculture & Life Sciences, University of Arizona, Tucson, Arizona, USA

**Keywords:** human papillomavirusE2, BRD4, ZC3H4, viral gene expression

## Abstract

High-risk human papillomaviruses (HPV), particularly HPV16, are major causes of anogenital and oropharyngeal cancers. The HPV late promoter, P670 in the case of HPV16, is activated upon host cell differentiation and drives the expression of viral capsid proteins. While differentiation-specific host transcription factors have been implicated in regulating this promoter, the mechanism remains incompletely understood. HPV E2 proteins activate transcription by interacting with the host protein BRD4 (Bromodomain-containing protein 4). A biotin proximity ligation screen identified several novel E2 interactors, of which many overlap with the BRD4 interactome, suggesting BRD4 mediates a large fraction of these interactions. One such interactor, ZC3H4 (Zinc finger CCCH domain-containing protein 4), is known to restrict the expression of long non-coding RNAs, including enhancer and promoter upstream antisense RNAs (uaRNAs). E2 recruits ZC3H4 in a BRD4-dependent manner to specifically activate the P670 promoter in reporter assays. Supporting this, E2 and ZC3H4 co-localize in cells with high P670 activity. ZC3H4 is upregulated during differentiation, and its knockdown in differentiated HPV16- or HPV31-positive cells reduces late viral transcripts in an E2-BRD4-dependent manner. Interestingly, knockdown of ZC3H4 does not increase viral uaRNAs, suggesting that ZC3H4 does not enhance HPV late transcription by regulating viral antisense transcription.

**IMPORTANCE:**

High-risk human papillomaviruses (HPVs), particularly HPV16, can cause anogenital and oropharyngeal cancers. HPV16 relies on the differentiation-dependent activation of its late promoter, P670, to produce capsid proteins. While host transcription factors contribute to this regulation, the mechanisms remain incompletely defined. Our findings reveal that the viral E2 protein collaborates with the host protein BRD4—a critical transcriptional regulator—to recruit other cellular partners, such as ZC3H4. Normally, ZC3H4 suppresses non-coding RNAs in cells, but HPV16 repurposes it via BRD4 to activate P670. This interaction intensifies in differentiated cells, where ZC3H4 levels rise, and disrupting ZC3H4 specifically blocks late viral gene expression without affecting antisense viral transcription. This highlights a unique, differentiation-dependent strategy HPV16 uses to hijack host machinery for its replication.

## INTRODUCTION

Persistent infections with high-risk (hr) human papillomaviruses (HPV), particularly HPV16, can cause anogenital and oropharyngeal cancers (1). HPVs replicate in keratinocytes, the primary cell type of cutaneous and mucosal epithelium, and have adapted their replication cycle to the differentiation state of the infected cell (2, 3). In basal-like keratinocytes, HPVs undergo a non-productive, persistent replication cycle, characterized by low levels of viral early gene expression and genome maintenance replication (2, 3). When infected cells move into the suprabasal layer, they start their terminal differentiation program, which is necessary for productive replication (2, 3). Productive replication starts in the upper spinous layer and is characterized by amplification of the viral DNA genomes and activation of the viral late promoter located in the *E7* gene, which is labeled P670 in HPV16 and P742 in HPV31 (4, 5). Activation of P670 results in high-level expression of a spliced *E1^E4, E5* transcript, which results in the abundant expression of the non-structural E4 protein, a marker for productive replication (6). Finally, P670 transcripts terminating at the late polyadenylation signal encode the L1 and L2 capsid proteins, and this enables the formation of infectious progeny virions in the granular layer of the epithelium, which are finally shed off into the environment (7).

In contrast to the major early promoter P97, responsible for the expression of the *E6* and *E7* oncogenes, the regulation of the late promoter is incompletely understood. The extrachromosomal maintenance of viral genomes is required for its activity upon differentiation (8). However, activation of the HPV31 late promoter P742 in differentiated cells also occurs when genome amplification is blocked (9, 10). This makes it likely that the differentiation-dependent activation of the late promoter precedes genome amplification. Late promoter activity is controlled by sequences in *E7* surrounding late promoter start sites and the viral upstream regulatory region (URR), which harbors transcriptional enhancer elements, the major early promoter, the replication origin, and DNA-binding sites for viral E1, E2, and E8^E2 proteins (9–11). While a large number of cellular transcription factors have been shown to bind to viral genomes in a differentiation-dependent manner (12, 13), only C/EBP-beta (CCAAT/enhancer-binding protein beta) and KLF4 (Krueppel-like factor 4) have been functionally implicated in controlling late promoter activity (14–16). Furthermore, HPV16 P670 has been shown to be activated in a differentiation-dependent manner at the level of transcriptional elongation which involves CDK8 (Cyclin-dependent kinase 8), CDK9 (Cyclin-dependent kinase 9), and BRD4 (11). CDK8 is a component of the mediator kinase module which controls RNA polymerase II transcription (17). CDK9 is part of the positive transcriptional elongation factor beta (P-TEFb) which is required for the transition from early to productive RNA polymerase II elongation (18). P-TEFb is recruited by BRD4 to promote phosphorylation of the C-terminal domain of RNA polymerase II (19).

The observation that replicating viral genomes, but not the amplification event *per se*, are required for late promoter induction in differentiating cells could indicate that extrachromosomal templates preferentially recruit host cell transcription factors required for late promoter activity or that viral proteins involved in genome replication also contribute to late promoter activity. The replication of HPV genomes is dependent upon the viral E1 and E2 proteins that form a high-affinity origin recognition complex which initiates replication upon binding (20). In addition, E2 has transcription-modulating activities (21). Upon overexpression, E2 can inhibit the viral major early promoter which depends on two proximal E2-binding sites (E2BS) overlapping with crucial promoter elements (21). HPV E2 also displays a potent transcriptional activation function, which is only evident with synthetic transcription units composed of multimerized E2BS and a minimal promoter (22). Both the transcription activation and repression functions of E2 depend greatly upon the interaction of conserved E2 residues with the cellular BRD4 protein (23–26). Interestingly, HPV31 mutant genomes encoding BRD4-binding impaired E2 only show reduced late gene transcription and viral genome amplification in differentiated cells (27, 28). One study reported that E2 does not contribute to P670 activity (29), but the reporter construct used lacked the URR and, thus, the conserved, high-affinity E2BS which are important for E2-mediated transcription activities (21). Furthermore, HPV16 E2 transcripts and protein amounts are increased in suprabasal, differentiated cells which display amplified viral genomes (30, 31). Consistent with this, a small fraction of cultured HPV16-positive cells displays nuclear HPV16 E2 accumulations which represent viral replication centers indicative for the viral productive phase (32). Taken together, a contribution of E2 to late promoter regulation is likely.

In addition to viral E1 and cellular BRD4 proteins, which are highly conserved interaction partners for all papillomavirus E2 proteins, a large number of E2-interacting host cell proteins have been identified using candidate, genetic, or immunoprecipitation-mass spectrometry approaches (21). Recently, biotin proximity ligation assays have been established in which the protein of interest is genetically fused to a biotin protein ligase which will, upon the addition of biotin, biotinylate proteins in close proximity *in vivo* that are either direct interaction partners of the protein of interest or within a distance of less than 10 nm (33). Biotinylated proteins are then identified by streptavidin enrichment followed by mass spectrometry. Using this approach in combination with different E2 proteins, we define an *in vivo* interactome for E2. The E2 interactome overlaps with a BRD4 interactome suggesting that many of the putative E2 interactors are recruited via BRD4. The host cell protein ZC3H4 (Zinc finger CCCH domain-containing protein 4) is present in both interactomes and was, therefore, further investigated. ZC3H4 is involved in the control of gene expression and is part of a checkpoint that promotes transcription termination of long non-coding (lnc), enhancer (e), and promoter upstream antisense (ua) RNAs and their subsequent degradation by the exosome (34–39). As these mechanisms might play a role in controlling HPV gene expression, ZC3H4 was further investigated. Using proximity ligation assay experiments, we find that HPV16 E2 and ZC3H4 interact *in vivo*, and this is dependent on the interaction of E2 with BRD4. HPV16 E2 in combination with ZC3H4 and BRD4 specifically activates P670 in reporter assays. Consistent with this, HPV16 E2 co-localizes strongly with ZC3H4 in viral replication centers when cells express E4 and, therefore, displays high P670 activity. ZC3H4 is induced by differentiation and a knock-down of ZC3H4 in differentiated HPV16 or 31-positive cells specifically reduces viral late transcripts, and this is dependent on the E2-BRD4 interaction. The knock-down of ZC3H4 in differentiated HPV16-positive cells induces cellular but not viral uaRNAs. Thus, the activation of viral late gene expression by ZC3H4 does not result from preventing viral antisense transcription in order to favor sense transcription. These data suggest a novel, activating function for ZC3H4 in conjunction with E2 and BRD4 in HPV-infected cells.

## RESULTS

### Proximity labeling reveals ZC3H4 as a putative interactor of E2

**Fig 1:**
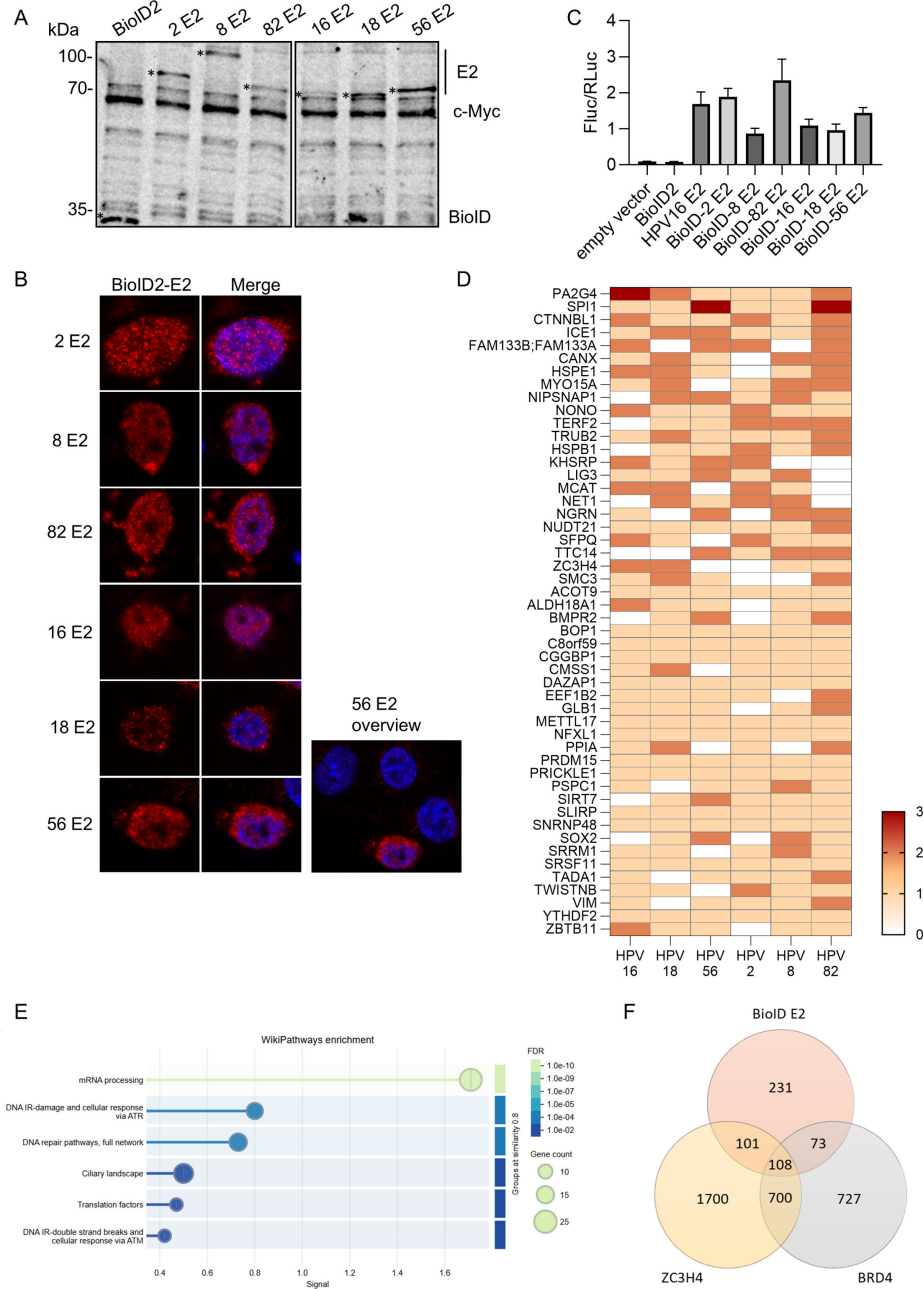
BioID2 proximity labeling reveals novel putative interaction partners of HPV E2 proteins. (**A**) C33A cells were transfected with the different BioID2-E2 expression vectors. After 48 h, cells were harvested and protein expression was analyzed by western blot using antibodies against the myc epitope which detects endogenous c-myc and myc-tagged BioID2-E2 proteins. BioID2 and E2-BioID2 fusion proteins are indicated by asterisks left to the lanes. (**B**) C33A cells were transfected with 100 ng of one of the BioID2-E2 expression plasmids and analyzed by immunofluorescence with anti-myc antibodies. DAPI was used to stain DNA. (**C**) C33A cells were transfected with 50 ng of pC18-Sp1-Luc, 10 ng of BioID2-E2 or pSG5 16 E2, and 0.5 ng of pCI-Neo RLuc as a transfection control. Values were calculated as the ratio of firefly to *renilla* luciferase. (**D**) Heat map illustrating the frequency with which each protein was significantly enriched relative to the negative control in three independent experiments. The top 50 hits from BioID-E2 proximity labeling experiments are shown. (**E**) A functional enrichment analysis (string-db.org) of proteins identified in the BioID-E2 proximity labeling experiment. Numbers in brackets represent the number of proteins in the respective category. (**F**) Comparison of the E2 proximity interactome with the BRD4 interactome extracted from the BioGrid database and the ZC3H4 proximity interactome published in reference 36.

To identify new potential cellular interactors of HPV E2 proteins, we performed a BioID-mass spectrometry experiment. We chose E2 proteins from HPV16 and 18 as the most carcinogenic alpha-HPV types from species α9 and α7, respectively, HPV56, a medium carcinogenic type from species α6, HPV82 a non-carcinogenic type from species α5, HPV2, a wart-causing type from species α2, and HPV8, which belongs to species β1 and is found in skin lesions of *Epidermodysplasia verruciformis* patients. BioID2-tagged E2 proteins of HPV 16, 18, 56, 2, 8, and 82 were expressed in HPV-negative C33A cells commonly used to investigate HPV E2 protein activities. Western blot experiments confirmed the expression of the different BioID2-E2 fusion proteins at slightly different levels (Fig. 1A). In addition, immunofluorescence analysis revealed a predominant nuclear localization of all BioID2-E2 fusion proteins consistent with the subcellular localization of wild-type E2 (Fig. 1B). Luciferase assays using the E2-dependent reporter plasmid pC18-Sp1-luc and untagged HPV16 E2 as a positive control revealed that all BioID2-E2 fusion proteins were able to activate the reporter plasmid to similar levels as wild-type HPV16 E2 (Fig. 1C). Taken together, these experiments indicate that BioID2-E2 fusion proteins are nuclear and exhibit transcription activation activity.

C33A cells were transfected with an expression vector encoding BioID2 only as a negative control, or the different BioID2-E2 fusions. Biotin-labeled proteins were isolated 48 h post transfection by streptavidin affinity purification and identified by mass spectrometry analysis. The enrichment of the biotinylated proteins from each of the different lysates with the BioID2-E2 fusion proteins was compared to the BioID2 only sample. In total, 514 proteins were significantly enriched at least once with any E2 protein. These included CPSF3/4, SF1, EP400, VIM, and C1QBP which have been previously identified as interactors of E2 proteins by different methods indicating the feasibility of the approach (40–43). The heat map shows the top 50 proteins identified in each of three independent experiments, along with the number of times each was enriched compared to the control (Fig. 1D). A functional enrichment analysis of all identified proteins using STRING (string-db.org) indicated an enrichment of mRNA processing and DNA repair and DNA damage pathways consistent with the involvement of E2 in transcription and DNA replication (Fig. 1E). While BRD4 was not identified in the screen, a comparison of reported BRD4 interactors using the BioGrid database (thebiogrid.org) with potential interactors from the BioID2-E2 screen revealed that 181 proteins were present in both data sets suggesting that many of the putative E2 interactors are recruited via BRD4 (Fig. 1F). We chose to follow-up on ZC3H4 as it was identified with HPV16 and 18 E2 proteins and is present in the BRD4 interactome. Furthermore, a comparison of the ZC3H4 (36), BRD4, and the biotin proximity interactomes of HPV E2 proteins revealed large overlaps between the three groups (Fig. 1F), suggesting that ZC3H4 could be important for E2-BRD4-mediated activities.

### ZC3H4 is in direct proximity to HPV16 E2

**Fig 2:**
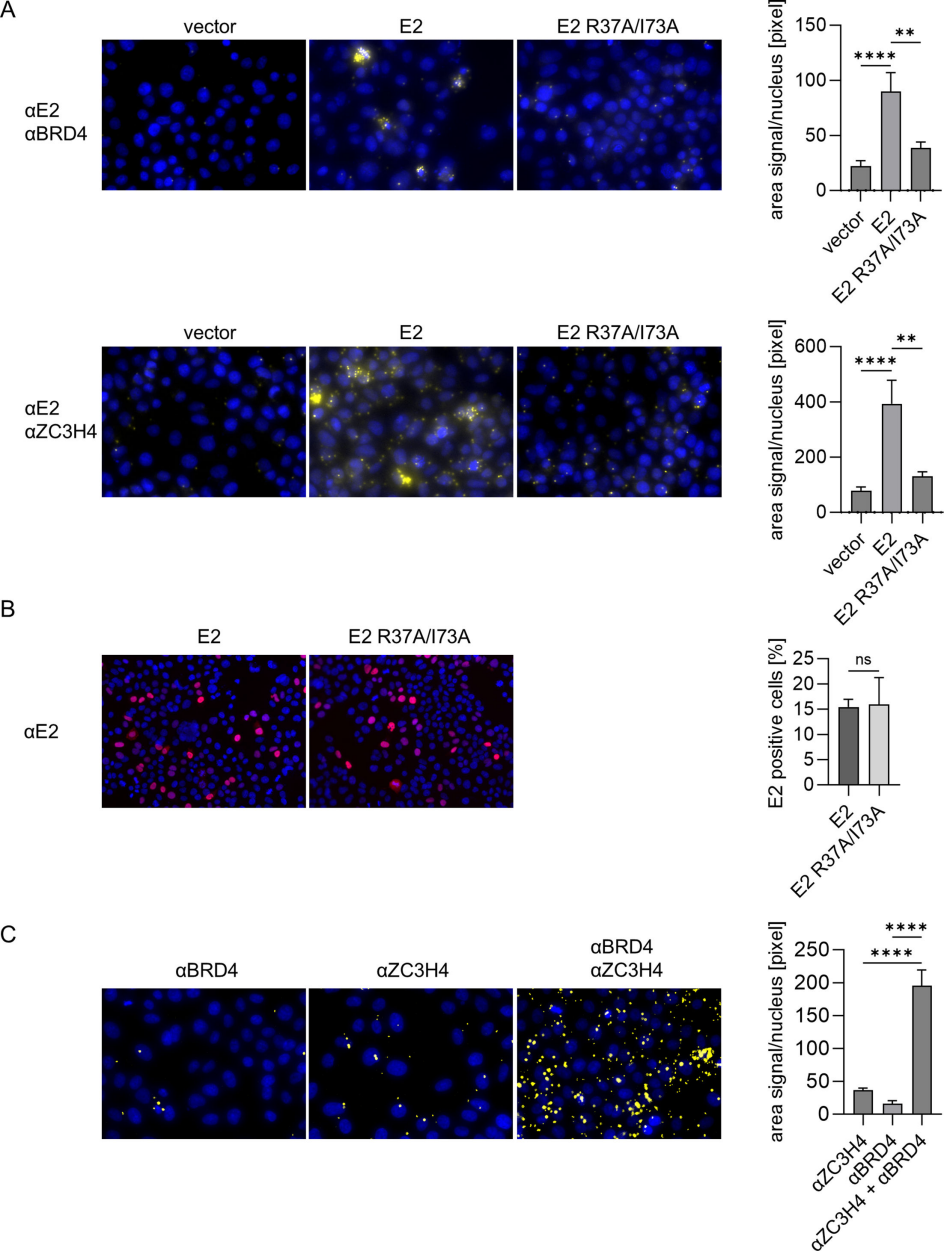
Proximity ligation assay (PLA) indicates a BRD4-depdent interaction of E2 and ZC3H4. (**A**) C33A cells were transfected with HPV16 E2 (25 ng; pSG5 16 E2-HA) or empty vector (25 ng; pSG5). Antibodies against HPV 16 E2, ZC3H4, or BRD4 were used. PLA signals were quantified by calculating the area of PLA signal per nucleus. PLA signals between E2 or HPV16 E2 R37A/I73A and BRD4 or ZC3H4 for in total *k* ≥ 50 nuclei in *n* = 3 experiments were determined, and mean values were calculated. Statistical significance was determined by one-way ANOVA and Dunnett’s multiple comparison test. **P < 0.01; ****P < 0.0001. (**B**) C33A cells were transfected with HPV16 E2 or HPV16 E2 R37A/I73A as described above and analyzed by immunofluorescence using the 22E2-B9 antibody. Random images were acquired, and between 424 and 767 nuclei were recorded in each of three independent experiments. The percentage of positive cells was determined for each experiment and then averaged. Statistical significance was determined by unpaired *t* test. Ns, *P* > 0.05. (**C**) PLA signals were quantified calculating the area of PLA signal per nucleus. PLA signals between BRD4 and ZC3H4 for a total *k* ≥ 54 nuclei in *n* = 3 experiments were determined, and mean values were calculated. Statistical significance was determined by one-way ANOVA and Dunnett’s multiple comparison test. ****P < 0.0001.

To validate the potential interactions between HPV16 E2, BRD4, and ZC3H4, a proximity ligation assay (PLA) was performed. We transfected HPV16 E2 into C33A cells and used antibodies against HPV16 E2 and ZC3H4, or HPV16 E2 and BRD4. In both cases, significantly higher PLA signals could be detected for both combinations compared to the negative control without HPV16 E2 (Fig. 2A). This indicates that E2 and ZC3H4 are in close proximity. Remarkably, the E2 mutant R37A/I73A, which is impaired in its interaction with BRD4 (23), not only significantly reduced the PLA signal with BRD4 but also with ZC3H4 (Fig. 2A). A control experiment revealed a similar number of HPV16 E2 wt and R37A/I73A mt-positive cells by indirect immunofluorescence suggesting that the differences in PLA signals between wt and mt E2 proteins are not caused by different protein expression levels (Fig. 2B). BRD4 gave rise to a robust PLA signal with ZC3H4 in the absence of E2 (Fig. 2C) confirming the BioGrid interaction data. Taken together, these data suggest that E2 is brought into close vicinity of ZC3H4 due to the mutual interaction of BRD4 with E2 and ZC3H4.

### HPV16 E2 activates the viral late P670 promoter in a BRD4- and ZC3H4-dependent manner

**Fig 3:**
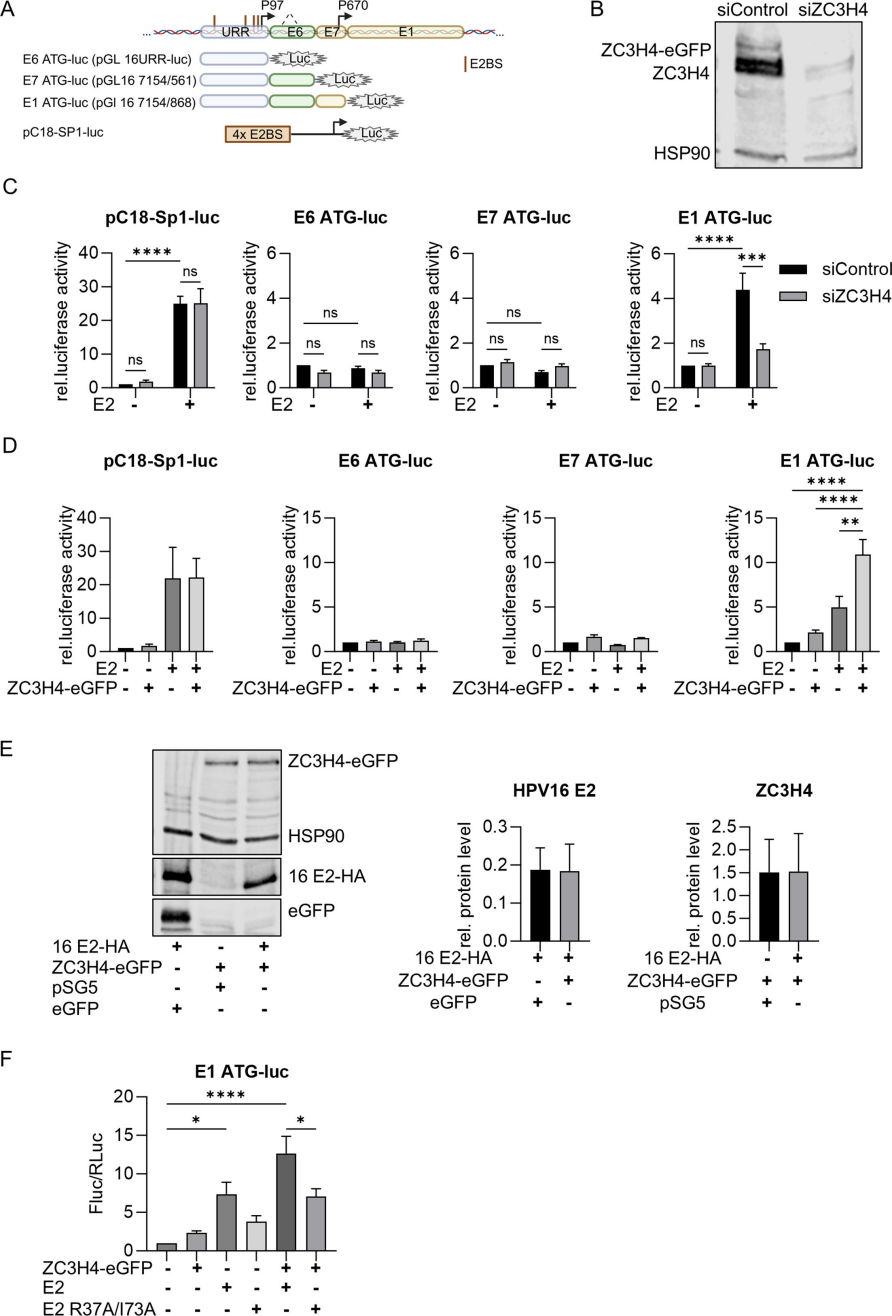
ZC3H4 increases late promoter activation by E2. (**A**) Schematic representation of the different HPV16 reporter constructs. Transcription start sites of the major early promoter P97 and late promoter P670 are indicated by arrows. (**B**) Immunoblot of C33A cells transfected with siRNA against ZC3H4 or a control siRNA and with 1,000 ng of ZC3H4-eGFP using antibodies specific for ZC3H4 to detect endogenous and transfected ZC3H4. HSP90 was used as a loading control. (**C**) C33A cells were treated with 15 pmol of siControl or siZC3H4 and 24 h later transfected with 0.5 ng pCI-Neo RLuc, 50 ng of one of the four reporter plasmids (pC18-Sp1-luc, E6 ATG-luc, E7 ATG-luc, E1 ATG-luc), 10 ng of pSG5 16 E2 or empty vectors. Values were calculated as the ratio of firefly to *renilla* luciferase activity. Mean values are derived from at least three independent experiments, and error bars indicate the SEM. Statistical significance was determined by one-way ANOVA and Dunnett‘s multiple comparisons test (compared to the activity of the luciferase reporter); **P < 0.01; ****P < 0.0001. (**D**) C33A cells were transfected with 0.5 ng pCI-Neo RLuc, 50 ng of one of the four reporter plasmids (pC18-Sp1-luc, E6 ATG-luc, E7 ATG-luc, E1 ATG-luc), 10 ng of 16 E2 and 50 ng of ZC3H4-eGFP or empty vectors. Values were calculated as the ratio of firefly to *renilla* luciferase activity and are expressed relative to the control. Mean values are derived from at least three independent experiments, and error bars indicate the SEM. Statistical significance was determined by one-way ANOVA and Dunnett‘s multiple comparisons test; ns *P* > 0.05; *P < 0.05, **P < 0.01. (**E**) C33A cells were transfected with 1,000 ng of 16E2-HA, 1,000 ng of ZC3H4-eGFP, or the corresponding empty pSG5 or eGFP expression plasmids. Forty-eight hours later, cells were harvested and protein levels measured via western blot, using anti-HA, anti-eGFP, and anti-HSP90 antibodies. Relative protein levels were calculated by normalizing to HSP90. (**F**) C33A cells were transfected with 0.5 ng pCI-Neo RLuc, 50 ng E1 ATG-luc, 10 ng of 16 E2 or 16 E2 R37A/I73A, and 50 ng of ZC3H4-eGFP or empty vectors in different combinations. Values were calculated as the ratio of firefly to *renilla* luciferase activity and are expressed relative to the control. Mean values are derived from at least three independent experiments, and error bars indicate the SEM. Statistical significance was determined by one-way ANOVA and Dunnett‘s multiple comparisons test; *P < 0.05, ****P < 0.0001.

Since ZC3H4, BRD4, and E2 are transcription regulators, transcription reporter assays were carried out using pC18-Sp1-luc, which can be strongly activated by E2 due to multimerized E2-binding sites close to the transcription start site, pGL16 URR in which the major early HPV16 promoter P97 drives luciferase expression from the E6 ATG, pGL16 7154/565, in which luciferase is expressed from the E7 ATG by P97, and pGL16 7154/868, in which luciferase is expressed from the E1 ATG (Fig. 3A). PGL16 7154/868 harbors, in addition to P97, the major late HPV16 promoter P670 (Fig. 3A). Furthermore, pGL16 7154/565 and pGL16 7154/868 contain the facultative introns in *E6* and, thus, can give rise to P97-initiated, spliced transcripts (Fig. 3A). For clarity reasons, the designations E6 ATG-luc for pGL16 URR, E7 ATG-luc for pGL16 7154/565, and E1 ATG-luc for pGL16 7154/868 will be used throughout the text. The different reporter constructs were co-transfected with an HPV16 E2 expression vector into C33A cells, and ZC3H4 protein expression was depleted by an siRNA targeting ZC3H4 (Fig. 3B). The pC18-Sp1-luc reporter was strongly activated by E2, and the knock-down of ZC3H4 had no effect on basal or E2-activated promoter activity (Fig. 3C). The knock-down of ZC3H4 had no effect on E6 ATG-luc or E7 ATG-luc in the absence or presence of E2 (Fig. 3C). Interestingly, E2 significantly activated E1 ATG-luc 4.8-fold, and this was reduced upon ZC3H4 knock-down (Fig. 3C).

To further analyze this, an expression plasmid for a GFP-ZC3H4 fusion protein was co-transfected with the different reporters in the presence and absence of HPV16 E2 (Fig. 3D). This revealed that ZC3H4 overexpression did not significantly influence pC18-Sp1-luc, E6 ATG-luc, or E7 ATG-luc in the absence or presence of E2 (Fig. 3D). In contrast, the activation of E1 ATG-luc by E2 was significantly increased by ZC3H4 overexpression, and this was not due to changes of E2 or ZC3H4 protein levels (Fig. 3D and E). The unique response of E1 ATG-luc to the combination of E2 and ZC3H4 suggested that they target the late P670 and not the early P97 promoter.

Since the E2-ZC3H4 interaction is BRD4-dependent and transcription activation by E2 is mainly mediated by the interaction with BRD4 (23, 24), we determined whether the activation of E1 ATG-luc by E2 and ZC3H4 is affected by the E2-BRD4 interaction. Compared to wild-type E2, the BRD4 binding-deficient HPV16 E2 R37A/I73A mutant showed a significantly reduced activation of E1 ATG-luc in the presence of over-expressed ZC3H4 (Fig. 3F). In the absence of additional ZC3H4, activation of E1 ATG-luc by E2 R37A/I73A was reduced, but this did not reach statistical significance. Taken together, these data suggest that E2 in conjunction with BRD4 and ZC3H4 activates the late HPV16 P670 promoter.

### ZC3H4 co-localizes with E2 foci in keratinocytes maintaining HPV16 genomes

**Fig 4:**
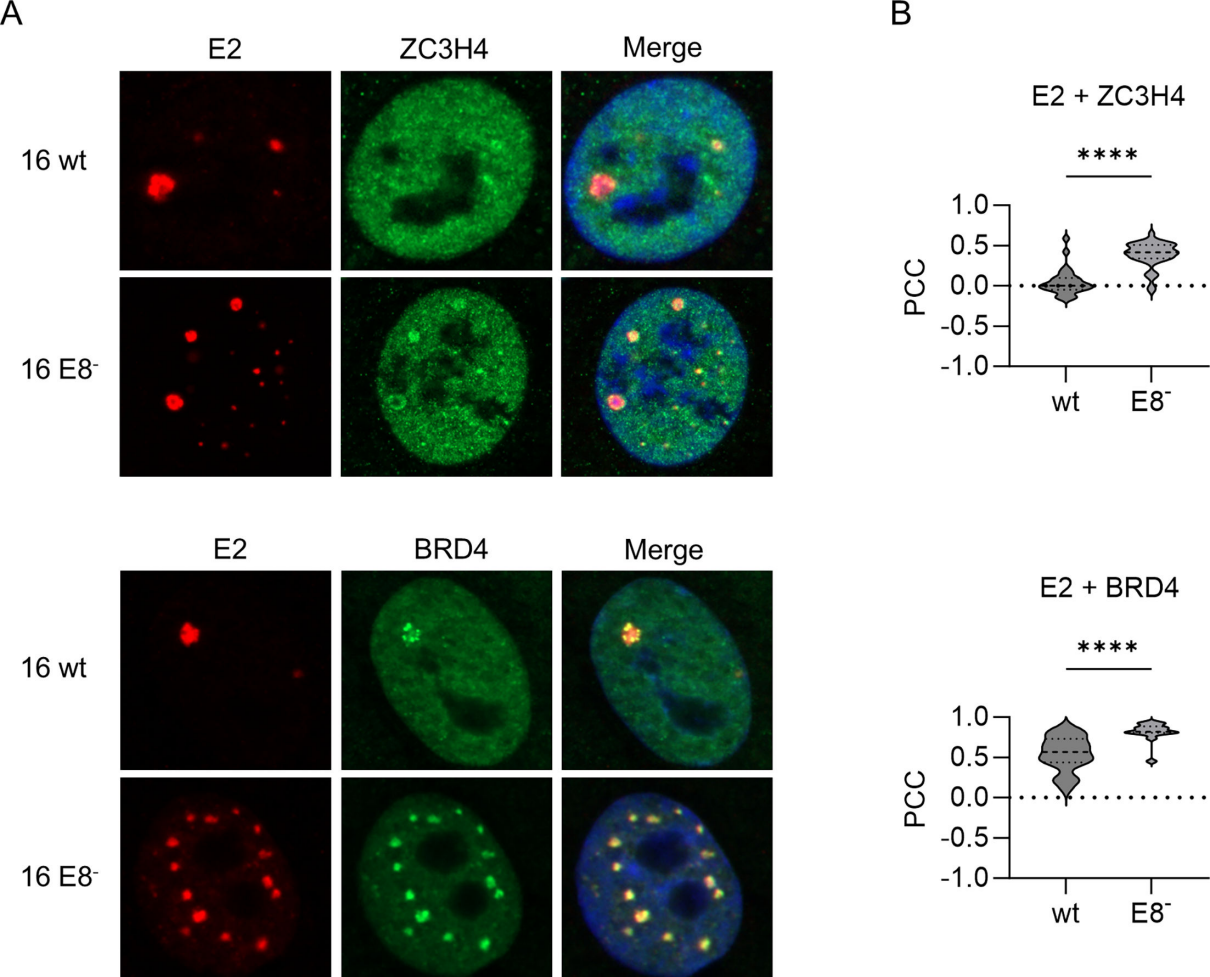
ZC3H4 is enriched in viral replication centers in HPV16 positive keratinocytes. (**A**) HPV16 wildtype or HPV 16 E8-positive keratinocytes were seeded and analyzed by immunofluorescence using antibodies against HPV16 E2, ZC3H4, and BRD4. DAPI was used to stain the DNA. (**B**) Colocalization between ZC3H4 and HPV16 E2 or HPV16 E2 and BRD4. The Pearson Correlation Coefficient for at least *n* ≥ 15 nuclei from two independent experiments was determined, and mean values were calculated. Statistical significance was determined by unpaired *t* test; ****P < 0.0001.

To explore the contribution of E2 and ZC3H4 to viral transcription in the context of replicating viral genomes, we used stable keratinocyte cell lines maintaining extrachromosomally HPV16 wt genomes. In addition, we used HPV16 E8- cell lines which cannot express the viral E8^E2 repressor protein and, therefore, have increased viral copy numbers and early and late gene expression (44). A recent study indicated that HPV16 E2 antibodies can detect defined nuclear E2 accumulations which represent viral replication centers indicating that productive replication has started in such cells (32). We have validated these findings by a set of different antibodies directed against HPV16 E2 which includes the previously described monoclonal antibody 22E2-B9 (45, 46) (manuscript in preparation). Immunofluorescence analysis of E2 and endogenous ZC3H4 in wt cells revealed low levels of co-localized ZC3H4 and E2 in E2-foci positive nuclei (mean PCC = 0.04; Fig. 4A and B). Interestingly, when these experiments were performed in E8-cells, which only express E2, co-localization between E2 and ZC3H4 (PCC = 0.45) was greatly increased in E2-foci (Fig. 4A and B). Remarkably, also an increase in the levels of co-localization of E2 with BRD4 from wt to E8- cells can be observed in these foci (Fig. 4A and B).

**Fig 5:**
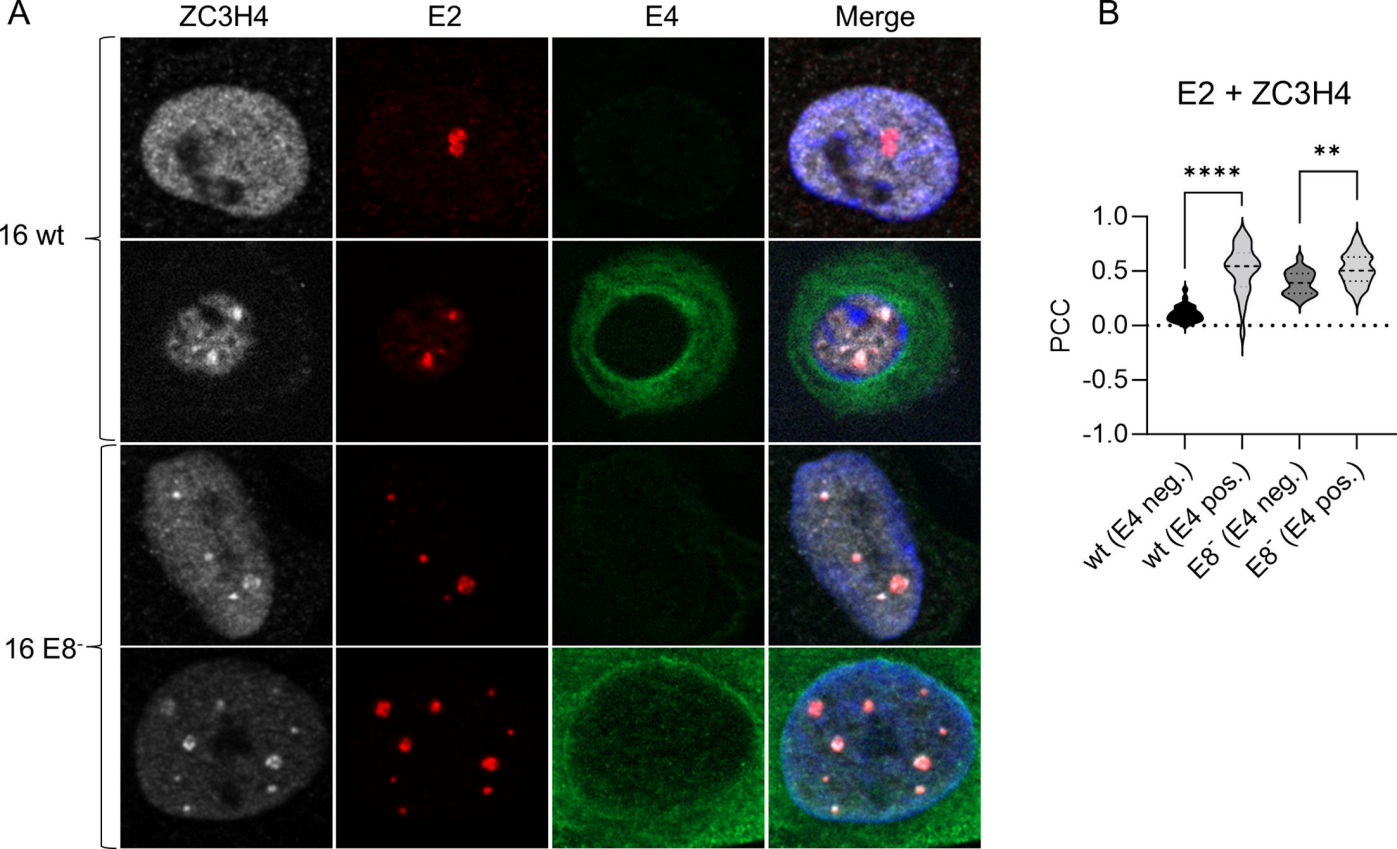
ZC3H4 is enriched in viral replication centers in HPV16 positive keratinocytes expressing the viral E4 protein. (**A**) HPV16 wildtype or HPV 16 E8-positive keratinocytes were seeded and analyzed by immunofluorescence using antibodies against 16 E2, ZC3H4, and 16 E4. DAPI was used to stain the DNA. (**B**) Colocalization between ZC3H4 and HPV16 E2 in HPV16 E4-positive or -negative cells. The Pearson Correlation Coefficient between E2 and ZC3H4 for *n* ≥ 21 nuclei from independent experiments was determined, and mean values were calculated. Statistical significance was determined by unpaired *t* test; **P < 0.01; ****P < 0.0001.

While characterizing E2 foci-positive cells in wt and E8-cell lines, we noted that only some express high levels of the viral late E4 protein (manuscript in preparation). Since E4 expression is driven by P670, such cells have high P670 activity. Interestingly, co-localization of E2 foci and ZC3H4 was significantly increased in E4-positive wt and E8-cells compared to E4-negative cells (Fig. 5A and B) Taken together, these data suggest that ZC3H4 is recruited by E2 in a BRD4-dependent manner into replication foci consistent with E2, BRD4, and ZC3H4 controlling late gene expression.

### Knockdown of ZC3H4 reduces viral late gene expression in differentiated keratinocytes

**Fig 6:**
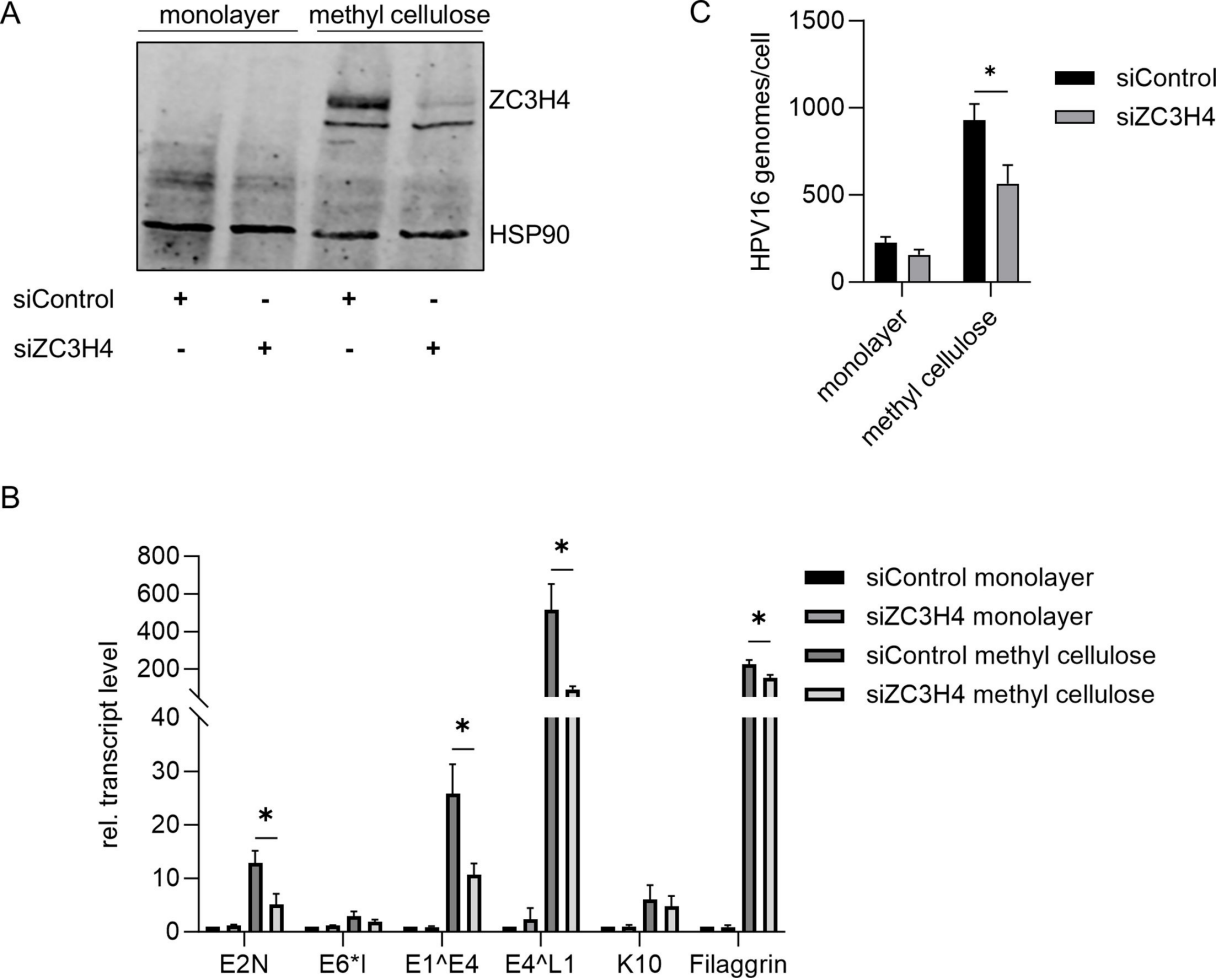
SiRNA-mediated knockdown of ZC3H4 decreases HPV16 late gene expression. (**A**) HPV16 wild-type keratinocytes were transfected with 75 pmol siZC3H4 or siControl and differentiated in methylcellulose medium for 48 h. Expression of endogenous ZC3H4 was analyzed by western blot using HSP90 as a loading control. (**B**) HPV16 wild-type keratinocytes were transfected with 75 pmol siZC3H4 or siControl and differentiated in methylcellulose for 48 h. Total RNA levels were measured by qPCR. Statistical significance was determined by one-way ANOVA and Dunnett‘s multiple comparisons test; *P < 0.05. (**C**) Viral genome copy numbers were calculated by qPCR after treatment with siZC3H4 or siControl and differentiation in methylcellulose, as described above. Statistical significance was determined by one-way ANOVA and Dunnett‘s multiple comparisons test; *n* = 3, *P < 0.05.

To address the function of ZC3H4 in the viral life cycle, we depleted ZC3H4 by siRNA in HPV16 wt in cell maintained in monolayer culture or differentiated in methyl cellulose-containing medium for 48 h. Immunoblot analysis revealed very low levels of ZC3H4 protein in undifferentiated cells that were greatly increased upon differentiation and efficiently reduced by the specific siRNA (Fig. 6A). Culture in methyl cellulose medium strongly induced the keratinocyte differentiation markers keratin 10 (*KRT10*) and filaggrin (*FLG*) (Fig. 6B). E6*I transcripts driven by the early P97 promoter were unchanged. *E2N*, spliced *E1^E4*, and spliced *E4^L1* transcripts were induced consistent with an activation of P670 by differentiation (Fig. 6B) (4). Knock-down of ZC3H4 significantly reduced *E1^E4*, *E2N*, and *E4^L1,* but not E6*I transcripts (Fig. 6B). Furthermore, no changes in *KRT10* and only a minor reduction of *FLG* transcripts were observed (Fig. 6B). These data suggest that ZC3H4 is specifically required for high-level transcription from P670 and not for the differentiation process. Differentiation resulted in an increase of viral genome copies, and this was also significantly reduced by knock-down of ZC3H4 (Fig. 6C). This could be a consequence of decreased *E2N* transcript levels which can encode E2 and, thus, would limit the amounts of E2 protein required for genome replication.

**Fig 7:**
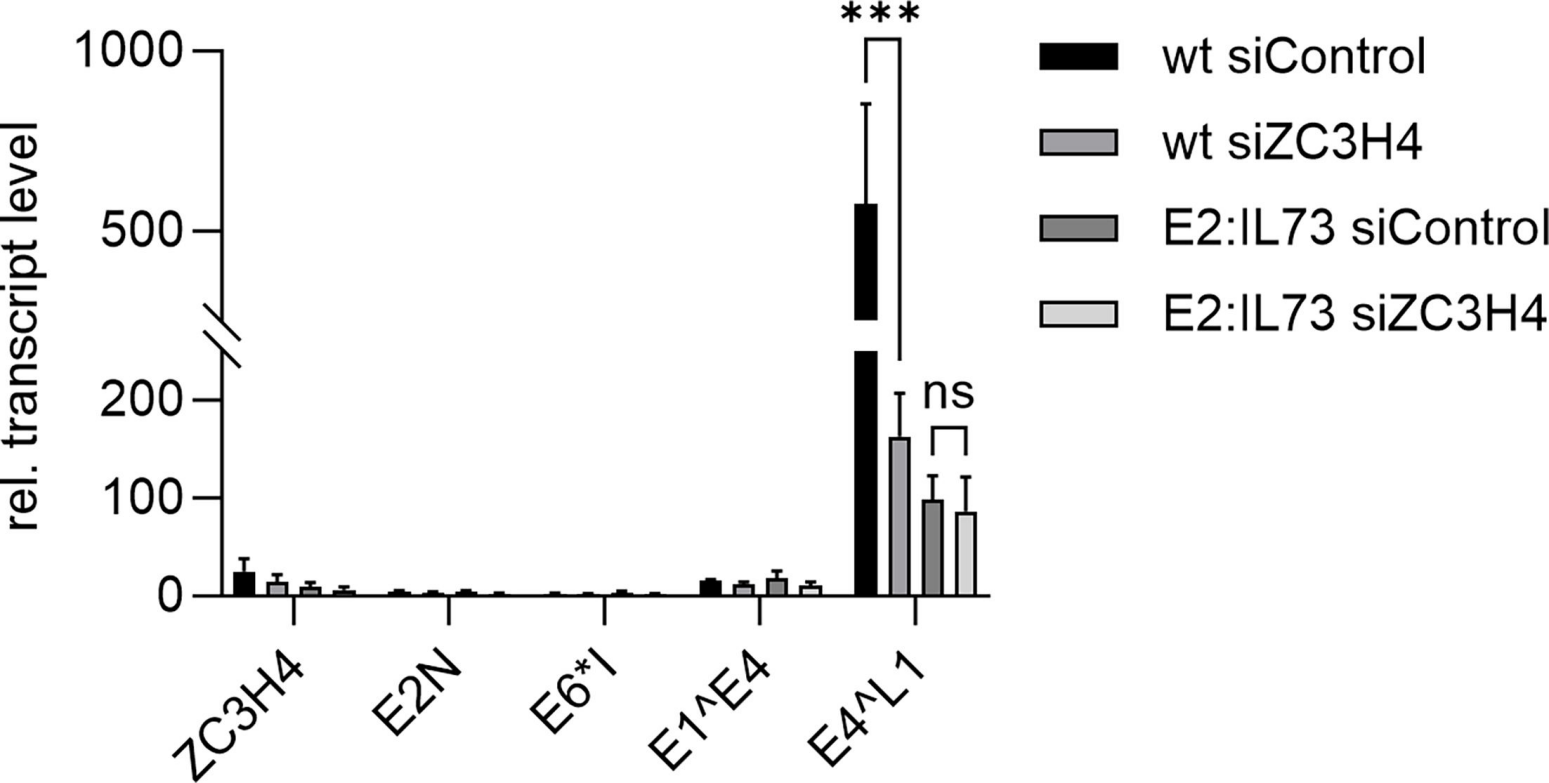
SiRNA-mediated knockdown of ZC3H4 decreases HPV31 late gene expression in wildtype, but not in E2:IL73-mutant cell lines. HPV31 wildtype or E2:IL73 positive keratinocytes were transfected with 75 pmol siZC3H4 or siControl and differentiated in methylcellulose medium for 48 h. Total RNA levels were measured by qPCR using *PGK1* as a reference gene. Statistical significance was determined by unpaired *t* test; *n* = 4 using three different donor cell lines, *P < 0.05.

To further explore the contribution of the E2-BRD4 interaction and ZC3H4 to late gene expression under physiological E2 protein levels expressed from replicating viral genomes, we used high risk HPV31. HPV31 is closely related to HPV16, but in contrast to HPV16, stable cell lines maintaining extrachromosomally replicating BRD4-binding deficient E2 genomes (HPV31 E2:IL73) can be established (28, 47–49). HPV31 wt or E2:IL73 keratinocyte lines were transfected with control siRNA or siZC3H4 and subjected to differentiation in methyl cellulose medium for 48 h. The late keratinocyte differentiation marker *FLG* was strongly induced in both cell lines compared to monolayer cultures indicating successful differentiation (Fig. 7). Differentiation also induced *ZC3H4* transcripts consistent with HPV16-positive cell lines (Fig. 6A and 7). Furthermore, *E4^L1* transcripts were strongly induced in HPV31 wt cell lines, and this was significantly reduced 3.5-fold by siZC3H4 consistent with HPV16 (Fig. 6B and 7). *E4^L1* transcripts were only induced 98-fold in E2:IL73 cell lines upon differentiation supporting the idea that the E2-BRD4 interaction activates late gene transcription in differentiated cells (Fig. 7). Remarkably, the knock-down of ZC3H4 did not significantly reduce *E4^L1* transcripts in E2:IL73 cell lines in contrast to wt cell lines (Fig. 7). These data are consistent with the idea that the E2-BRD4 interaction recruits ZC3H4 in order to achieve high level late gene expression in differentiated cells.

### Knockdown of ZC3H4 does not increase HPV16 antisense transcription in differentiated keratinocytes

**Fig 8:**
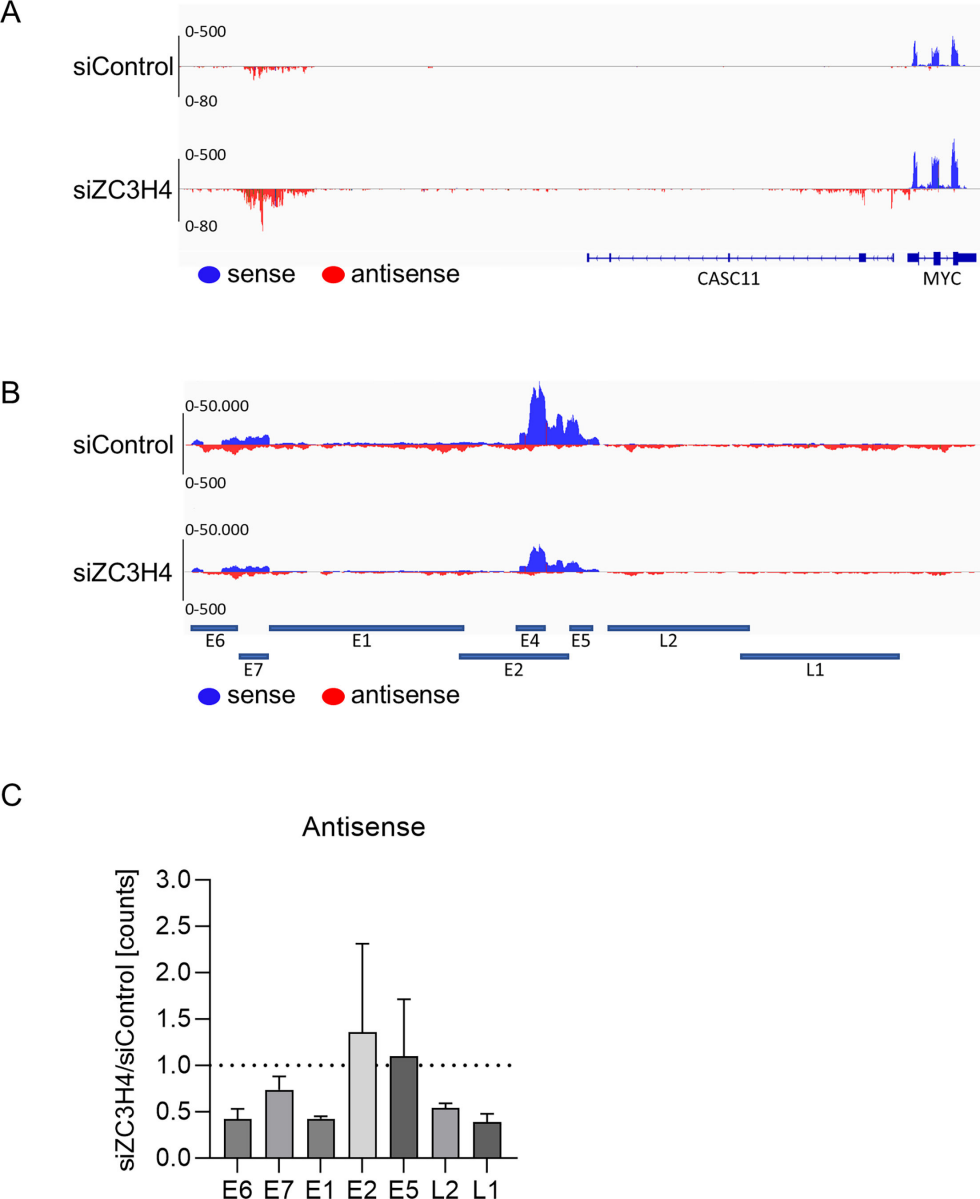
Strand-specific RNA-seq after ZC3H4 knockdown. RNA samples (*n* = 3) from differentiated HPV16 positive keratinocytes described above (Fig. 6B) were used for strand-specific RNA sequencing. (**A**) IGV plot of the upstream region of the MYC gene shown previously to be regulated by ZC3H4 (36). (**B**) IGV plot of the HPV16 genome. (**C**) Viral mRNA counts after ZC3H4 knockdown normalized to siControl in antisense direction.

ZC3H4 regulates gene expression and functions as part of a checkpoint that facilitates the transcription termination of lncRNAs, eRNAs, and promoter uaRNA (34–39). In line with this, knockdown of ZC3H4 results in increased lncRNA, eRNA, and promoter uaRNA transcription in human cell lines (36). All conserved PV genes are encoded by the sense strand of the viral genome, and there is currently little evidence for robust antisense transcription. Nevertheless, antisense HPV transcription could be inhibited by ZC3H4 during productive replication, and this might contribute to the activation of late gene transcription. To test this, RNA samples of differentiated HPV16 wt keratinocytes transfected with control siRNA or siZC3H4 analyzed in Fig. 6 were subjected to strand-specific RNA-sequencing. We first examined the region upstream of the *MYC* gene described to display increased uaRNA transcription upon ZC3H4 knock-down as a positive control (36). Consistent with this, ZC3H4 knockdown resulted in an upregulation and extension of uaRNA transcripts upstream of *MYC* (Fig. 8A). Very low levels of antisense transcription compared to sense transcription can be observed throughout the HPV16 genome in siControl transfected cells (Fig. 8B, please note the different scales for sense and antisense transcripts). Interestingly, no increase of antisense transcription upon ZC3H4 knock-down is visible upstream of the major late P670 nor of the major early P97 promoter (Fig. 8B). Furthermore, quantification of antisense transcription in the *E6*, *E7*, *E1*, *E2*, *E5*, *L2*, and *L1* genes rather indicated decreased levels in *E6*, *E7*, *E1*, *L2,* and *L1* and only very slightly increased levels in *E2* and *E5* (Fig. 8C). These data indicate that the antisense strand of HPV16 is not robustly transcribed in differentiated cells and that this is not counteracted by ZC3H4. In summary, the data do not support the idea that ZC3H4 controls HPV16 late gene expression by preventing viral antisense transcription.

## DISCUSSION

A key step in the HPV16 replication cycle is the activation of the late promoter P670 in differentiated keratinocytes, which drives the expression of the L1 and L2 capsid proteins required for the production of infectious virions. Previous reports had indicated that differentiation-regulated cellular transcription factors such as C/EBP-beta activate the HPV late promoter (14, 16). However, extrachromosomally replicating genomes are also required for late promoter induction pointing to the possibility that viral replication factors such as E1 or E2 might also play a role (8). A prime candidate is E2, which not only activates viral replication through its interaction with E1 but is also a potent transcription activator through its interaction with BRD4 (23, 24).

Our biotin proximity ligation screen of HPV E2 proteins from different HPV types identified a large number of host cell proteins as putative, novel interactors (Fig. 1). While we did not identify BRD4 in this screen, we observed a large overlap of the BRD4 interactome and the E2 biotin proximity interactome making it likely that many of these factors are recruited via BRD4. We focused on ZC3H4 as it was identified in the proximity interactome of the E2 proteins of the major high risk HPV16 and 18, is part of the BRD4 interactome, and has been implicated in the control of cellular gene expression by regulating pervasive transcription. Proximity ligation assays indicated that both E2 and BRD4 interact with ZC3H4. However, the ZC3H4 interaction depends on E2 binding to BRD4 as the BRD4-binding deficient E2 mutant R37A/I73A failed to interact (Fig. 2) which is consistent with ZC3H4 being indirectly recruited to E2 via BRD4.

Reporter assays in the C33A cell line, an HPV-negative cervical cancer cell line, revealed that E2 can activate the HPV16 late promoter P670 and that this is enhanced by ZC3H4. Co-localization analyses in cell lines maintaining replicating HPV16 genomes indicated that E2 and ZC3H4 are in close proximity in viral replication centers visualized by nuclear E2 foci, and this was especially pronounced in E2 foci formed by genomes that only express E2 or in E2 foci-positive wt cells when the late E4 protein is expressed, and thus, the viral late promoter P670 is highly active (Fig. 4 and 5). Consistent with viral late gene transcription being specifically activated by ZC3H4, a knock-down of ZC3H4 in differentiated HPV16 wt cells predominantly reduces transcripts that are mainly derived from the late P670 promoter, but not from the early P97 promoter. Previous studies revealed that HPV31 E2 I73L mt genomes, encoding a BRD4-binding and, therefore, transactivation-impaired E2, display reduced *L1* transcript levels in organotypic cultures (27, 28). Knocking down ZC3H4 in differentiated HPV31 wt or E2:IL73 cell lines reduces *E4^L1* transcripts only in wt cells consistent with a functional interaction of E2, BRD4, and ZC3H4 to activate late transcription. Interestingly, ZC3H4 expression increases upon differentiation. Taken together, these data strongly suggest that E2 binds to BRD4, which, in turn, recruits ZC3H4 to viral genomes in the productive phase to activate late transcription. Previous studies have indicated that P670 is regulated by transcriptional elongation and this involves CDK9, a component of the transcription elongation factor P-TEFb, BRD4, and CDK8, a component of the mediator complex (11). BRD4 interacts with P-TEFb to facilitate transcriptional elongation, and E2 has been shown to recruit P-TEFb via BRD4 to activate transcription of E2-dependent promoters (50). Consistent with this, the P-TEFb components CDK9 and cyclin T1 were also identified in the E2 biotin proximity interactome, albeit at a very low frequency. Thus, it is likely that E2 recruits P-TEFb to the late promoter and enables transcriptional elongation. In line with this, the short form of BRD4, BRD4S, which also interacts with E2 but cannot recruit P-TEFb as it lacks the C-terminal motif, is an inhibitor of HPV31 late transcription (26, 51–53). Since we used an antibody specific for the long form of BRD4 in the PLA assays and co-localization studies, it is unlikely that BRD4S recruits ZC3H4 to E2. Thus, BRD4S might inhibit viral late gene transcription by preventing the recruitment of BRD4-specific transcription activators such as P-TEFb and ZC3H4.

The activity of the HPV16 major early P97 promoter in the presence of E2 was not further modulated by ZC3H4. Consistent with this, levels of P97-derived E6* transcripts expressed from replicating HPV16 or 31 genomes were unaffected by the knock-down of ZC3H4. Interestingly, a synthetic E2-activated reporter construct, while being highly dependent upon recruitment of BRD4 by E2 (23, 54), did not respond to ZC3H4 depletion or overexpression, suggesting that E2 activation is not always ZC3H4-dependent. This suggests that the activation by E2, BRD4, and ZC3H4 is specific for P670. ZC3H4 restricts non-coding RNA transcription including lncRNA, eRNA, and uaRNA (34–39). ZC3H4 also regulates protein-coding RNAs, but the underlying mechanism has not been resolved (36–38). It has been suggested that ZC3H4 is a major contributor to transcription directionality, as a proportion of RNA polymerase II moves in the opposite direction and produces antisense instead of protein-coding, sense transcripts in the absence of ZC3H4 (38). Strand-specific RNA-seq analyses of differentiated HPV16 cell lines upon ZC3H4 knock-down revealed an increase of antisense transcription at the *MYC* gene consistent with published data (36) but failed to detect an increase of HPV16 antisense transcription upstream of P670 or elsewhere in the viral genome. Thus, the model that ZC3H4 prevents viral antisense transcription to increase viral sense transcription does not apply for HPV16.

ZC3H4 enhances HPV late promoter activity, whereas it mainly represses cellular pervasive transcription. The increase of uaRNA upstream of the *MYC* gene upon knock-down of ZC3H4 in differentiated HPV16-positive keratinocytes is similar to HPV-negative cells (36) making it unlikely that ZC3H4 activity is altered by HPV. Thus, it is most likely the specific combination of viral and cellular factors assembling on HPV late promoters that create an environment in which ZC3H4 specifically activates transcription.

## MATERIALS AND METHODS

### Recombinant plasmids

The Myc-BioID2-MCS expression vector, a gift from Kyle Roux (Addgene plasmid # 74223; https://www.addgene.org/74223/; RRID:Addgene_74223 [55]), was modified by inserting a Kpn2I restriction site at nt. 1654 resulting in Myc-BioID2-MCS (Kpn2I). The E2 genes of HPV2, 8, 16, 18, and 82 or a codon-optimized version of HPV56 E2 (GenScript) were inserted between the Kpn2I and BamHI restriction sites of Myc-BioID2-MCS (Kpn2I). Plasmids pGL16 URR (E6 ATG-luc), pGL16 7154/868 (E1 ATG-luc), pSG16 E2, pSG16 E2-HA, and pSG16 E2 R37A/I73A have been previously described (44, 54, 56). The pGL16 7154/561 (E7 ATG-luc) expression plasmid was constructed by replacing a BmgBI/ NcoI-fragment in pGL16 7154/868 with a PCR-fragment that introduced a NcoI restriction site in front of the HPV16 E7 ATG, which results in the insertion of a C after nt. 561 not encoded by HPV16. Plasmid pCI-neo-Rluc expresses *renilla* luciferase (*Renilla reniformis*) and has been previously described (57). The ZC3H4- pcDNA3.1(+)-C-eGFP expression vector was kindly provided by Steven West (36).

### Cell culture

C33A cells were maintained in Dulbecco’s modified Eagle’s medium supplemented with 10% fetal bovine serum and antibiotics. Stable HPV16 wt, HPV16 E8-, HPV31 wt, and HPV31 E2:IL73 keratinocyte lines were maintained in E-medium/10%FBS/penicillin-streptomycin in the presence of mitomycin C-arrested NIH 3T3 J2 mouse fibroblast as previously described (28, 44). HPV-positive keratinocytes were induced to differentiate by culture in methylcellulose-medium as previously described (58).

### Proximity ligation assay

C33A cells were transfected with HPV16 E2 or the empty vector pSG5. PLA staining was performed using a NaveniFlex Cell MR Red kit (Navinci) following the manufacturer’s instructions. The following antibodies were used: HPV16 E2 (recombinant mouse monoclonal, 22E2-B9; 1:200 [46]), anti-ZC3H4 (rabbit polyclonal, Sigma-Aldrich; HPA040934; 1:100), anti-BRD4 (rabbit monoclonal, Cell Signaling; E2A7X; 1:1,000, [specific for the long isoform of BRD4]), anti-BRD4 (mouse monoclonal, Sigma-Aldrich; AMAb90841; 1:200). Signals were detected using Cytation3 plate reader (BioTeK). Pictures were analyzed and quantified with ImageJ (Fiji) software.

### Luciferase reporter assays

C33A cells (7.5 × 10^4^) were transfected using Fugene HD (Promega) and the DNA amounts indicated in the figure legends. For siRNA knockdown experiments, C33A cells (6 × 10^4^) were transfected first with siRNA using Lipofectamine RNAiMAX Transfection Reagent (Invitrogen) as indicated in the figure legends and then with DNA using Fugene HD 24 h later. Cells were lysed in 150 µL of lysis buffer (100 mM K-PO_4_ [pH 7.8], 1% (vol/vol) Triton-X-100, 1 mM DTT) for 10 min on ice and firefly and *renilla* luciferase activities were determined with the respective substrates 48 h post DNA transfection using a TriStar2 S LB 942 multimode plate reader (Berthold Technologies). Firefly luciferase activities were normalized to *renilla* luciferase activities.

### siRNA

To target human ZC3H4, ON-TARGETplus Human ZC3H4 (23211) siRNA—SMARTpool (Dharmacon, Thermo Scientific; L-023865-02-0005) was used.

### RNA analysis

Total RNA was isolated from cultured keratinocytes using the RNeasy minikit (Qiagen). RNA was reverse transcribed using the QuantiTect reverse transcription kit (Qiagen), and cDNA was analyzed by qPCR in a LightCycler 480 (Roche Applied Science) using LightCycler 480 SYBR green I master mix (Roche Applied Science) and primers shown in Table 1. LightCycler 480 software (version 1.5) was used for quantification. The second derivative/max analysis was chosen to obtain Cq values, and melting curve analysis was used to ensure measurement of a single amplicon.

**TABLE 1:** Primer sequences used for quantitative PCR^*a*^

Use	Gene	Sequence (5′−3′)	Direction	Reference
Viral copy number	*ACTB*	GATATCGCCGCGCTCGTCGTCGAC	Forward	(44)
		CAGGAAGGAAGGCTGGAAGAGTGC	Reverse	(44)
	*HPV16 E6*	GAGAACTGCAATGTTTCAGGACC	Forward	(44)
		TGTATAGTTGTTTGCAGCTCTGTGC	Reverse	(44)
mRNA levels	*HPV16 E1^E4*	AGGCGACGGCTTTGGTATG	Forward	(59)
		TGGCTGATCCTGCAGCAGC	Reverse	(59)
	*HPV16 E4^L1*	CCCTGCCACACCACTAAGTT	Forward	(60)
		CTGGGACAGGAGGCAAGTAG	Reverse	(60)
	HPV16 E6*I	ACAGTTACTGCGACGTGAGATG	Forward	(59)
		TTCTTCAGGACACAGTGG	Reverse	(59)
	*PGK1*	CTGTGGGGGTATTTGAATGG	Forward	(44)
		CTTCCAGGAGCTCCAAACTG	Reverse	(44)
	*Keratin 10*	CGCCTGGCTTCCTACTTGG	Forward	(60)
		CTGGCGCAGAGCTACCTCA	Reverse	(60)
	*Filaggrin*	GACATGGCAGCTATGGTA	Forward	
		AATCCCAGTTGTTTCGATA	Reverse	
	*ZC3H4*	AATCCAAGCACAAACGCCAT	Forward	
		TCGTAGTCCTCGTACATGCC	Reverse	
	HPV31 E6*	AATTGTGTCTACTGCAAAGGTGTA	Forward	(61)
		CCAACATGCTATGCAACGTC	Reverse	(61)
	*HPV31 E1^E4*	TGTTAATGGGCTCATTTGGAA	Forward	(61)
		GGTTTTGGAATTCGATGTGG	Reverse	(61)
	*HPV31 E2N*	CTGTTGTGGAAGGGCAAGTT	Forward	
		TCCCAGCAAAGGATATTTCG	Reverse	
	*HPV31 E4^L1*	CATGCACAAACCAAACAAGG	Forward	(61)
		GCACTGCCTGCGTGATAATA	Reverse	(61)

^*a*^ Sequences of forward and reverse primers used for quantitative PCR are shown.

### Analysis of viral copy number

Total cellular DNA was isolated from HPV16 positive keratinocytes using DNeasy Blood & Tissue Kit (Qiagen). DNA was analyzed by qPCR in a LightCycler 480 (Roche Applied Science) using LightCycler 480 SYBR green I master mix (Roche Applied Science). Copy numbers were determined by known plasmid standards analyzed in parallel. The standard curve for HPV16 *E6* was generated using serial dilutions of HPV16 genomic DNA. The standard curve for *ACTB* was generated using serial dilutions of keratinocyte genomic DNA.

### Immunoblot analysis

To validate the expression of the fusion proteins, 3  ×  10^5^ C33A cells were seeded in 6-well dishes and transfected with 1 µg DNA and FuGENE HD (Promega). Cells were harvested 48 h post transfection and lysed in RIPA (1% [vol/vol] IGEPAL CA-630, 1% [wt/vol] sodium deoxycholate, 0.1% [wt/vol] SDS, 150 mM NaCl, 10 mM sodium phosphate pH 7.2, 2 mM EDTA, 50 mM sodium fluoride, 1× cOmplete protease inhibitor (EDTA free), 1× PhosStop phosphatase inhibitor) buffer. Immunoblot analysis was carried out as previously described (53). To control the knock-down of endogenous proteins, 4  ×  10^5^ HPV16 positive keratinocytes were seeded in 6-well dishes and transfected with siRNA. The next day, cells were trypsinized and one part was differentiated in methylcellulose. The other part of the cells was seeded in new 6-well dishes and maintained as a monolayer. The following primary antibodies were used: HA-Tag rabbit MAb (Cell Signaling; C29F4; 3724S; 1:1,000), anti-ZC3H4 (Sigma-Aldrich; HPA040934; 1:1,000), anti-c-Myc MAb (Santa Cruz; sc-40; 1:1,000), anti-eGFP mouse MAb (Santa Cruz; sc-9996; 1:1,000), HSP90 mouse MAb (Santa Cruz; sc-69703; 1:1,000). Bound antibodies were detected with IRDye 680RD goat anti-rabbit IgG (926-68071), IRDye 800CW goat anti-mouse IgG (926-32210) at a 1:15,000 dilution. Antibodies were diluted in PBS/0.1% Tween 20, and washing steps were performed with PBS/0.1% Tween 20. Fluorescent signals were recorded with a LI-COR Odyssey Fc (LI-COR Biosciences).

### Immunofluorescence analysis

For immunofluorescence analyses, cells were seeded on 35 mm glass bottom dishes (MatTek Life Sciences) and transfected the next day with expression plasmids. Cells were fixed 48 h later by the addition of 4% paraformaldehyde and incubated for 15 min at room temperature. Afterward, cells were washed three times with PBS and incubated at RT for 1 h in blocking buffer (1× PBS, 5% [vol/vol] normal goat and donkey serum, 0.3% [vol/vol] Triton-X 100) followed by incubation in primary antibody diluted in antibody-dilution-buffer (1× PBS, 1% [wt/vol] BSA Fraction V, 0.3% [vol/vol] Triton-X 100) at 4°C overnight. The next day, dishes were washed three times in PBS and then incubated with the appropriate secondary fluorescence labeled antibodies (goat anti-mouse-Alexa Fluor Plus 488 [Thermo Fisher Scientific A32723]; donkey anti-rabbit- Alexa Fluor Plus 488 [Thermo Fisher Scientific A32731]; donkey anti-mouse-Alexa Fluor Plus 555 [Thermo Fisher Scientific A32773]; goat anti-rabbit- Alexa Fluor Plus 555 [Thermo Fisher Scientific A21428]); goat anti-mouse-Alexa Fluor 488 IgG1 (y1) (Thermo Fisher Scientific A21121); goat anti-mouse-Alexa Fluor 555 IgG2a (y2a) (Thermo Fisher Scientific A21137); donkey anti-rabbit-Alexa Fluor 647 (Thermo Fisher Scientific A31573) for 1 h at room temperature. After three PBS washing steps, DAPI solution was added for 30 s to stain DNA, followed by three additional washing steps with PBS. Samples were analyzed with a Zeiss Axio Observer microscope and the appropriate filter sets. 63× magnification was used in combination with a Zeiss Apotome. The following antibodies were used for analysis: HA-tag (rabbit monoclonal, Cell Signaling; 3724S; 1:800), HA-tag (mouse monoclonal, Cell Signaling; 2367S; 1:100), anti-c-myc (mouse monoclonal, Santa Cruz; sc-40; 1:500), HPV16 E2 (recombinant mouse monoclonal IgG2, 22E2-B9; 1:200 [46]), HPV16 E4 (mouse monoclonal IgG1, Santa Cruz, sc-53324; 1:100) and anti-ZC3H4 (rabbit polyclonal, Sigma-Aldrich; HPA040934; 1:100), anti-BRD4 (rabbit monoclonal, Cell Signaling; E2A7X; 1:1,000, [specific for the long isoform of BRD4]), or anti-BRD4 (mouse monoclonal, Sigma-Aldrich; AMAb90841; 1:200).

### Biotin proximity ligation screen

C33A cells (4 × 10^6^) were seeded in 150 mm cell culture dishes and transfected with 10 µg of BioID2-E2 expression vector or a BioID2 expression vector as control. Twenty-four hours later, 50 µM Biotin was added to cell media. Cells were lysed with lysis buffer (50 mM Tris-HCL [pH 8], 400 mM NaCL, 0.3% [vol/vol] IGEPAL CA-630, 1 mM DTT, 1× cOmplete protease inhibitor [EDTA-free], 1× PhosStop phosphatase inhibitor). Biotinylated proteins were then enriched by using Streptavidin Sepharose (IBA Lifesciences). Fifty microliters of packed beads per sample was washed with TBS and lysis buffer (TBS) containing 0.5% IGEPAL CA-630, complete protease inhibitors (Roche), and phosphatase inhibitor cocktails 2 and 3 (Sigma). Equal amounts of lysate were then added to the beads, and the mixture was incubated for 1 h at 4°C under constant agitation. Following incubation, the beads were washed three times with TBS before they were subjected to an on-bead digestion.

### Mass spectrometry

Affinity-purified proteins were eluted by on-bead digestion as described previously (62). LC-MS/MS analysis was performed on Ultimate3000 nanoRSLC systems (Thermo Scientific) coupled to an Orbitrap Fusion Tribrid mass spectrometer (Thermo Scientific) by a nano spray ion source. Tryptic peptides were loaded onto a µPAC Trapping Column with pillar diameter of 5 µm, inter-pillar distance of 2.5 µm, pillar length/bed depth of 18 µm, external porosity of 9%, bed channel width of 2 mm, and length of 10 mm; pillars are superficially porous with a porous shell thickness of 300 nm and pore sizes in the order of 100–200 Å at a flow rate of 10 µL per min in 0.1% trifluoroacetic acid in HPLC-grade water. Peptides were eluted and separated on the PharmaFluidics µPAC nano-LC column: 50 cm µPAC C18 with a pillar diameter of 5 µm, inter-pillar distance of 2.5 µm, pillar length/bed depth of 18 µm, external porosity of 59%, bed channel width of 315 µm, and bed length of 50 cm; pillars are superficially porous with a porous shell thickness of 300 nm and pore sizes in the order of 100–200 Åby a linear gradient from 2% to 30% of buffer B (80% acetonitrile and 0.08% formic acid in HPLC-grade water) in buffer A (2% acetonitrile and 0.1% formic acid in HPLC-grade water) at a flow rate of 300 nL/min over 117 min. Remaining peptides were eluted by a short gradient from 30% to 95% buffer B in 5 min. Analysis of the eluted peptides was done on an LTQ Fusion mass spectrometer. From the high-resolution MS pre-scan with a mass range of 335–1,500, the most intense peptide ions were selected for fragment analysis in the orbitrap depending by using a high-speed method if they were at least doubly charged. The normalized collision energy for HCD was set to a value of 27, and the resulting fragments were detected with a resolution of 120,000. The lock mass option was activated; the background signal with a mass of 445.12003 was used as lock mass (63). Every ion selected for fragmentation was excluded for 20 s by dynamic exclusion. MS/MS data were analyzed using the MaxQuant software (version 1.6.1.0) (64, 65). As a digesting enzyme, Trypsin/P was selected with maximal 2 missed cleavages. Cysteine carbamidomethylation was set for fixed modifications, and oxidation of methionine and N-terminal acetylation were specified as variable modifications. The data were analyzed by label-free quantification with the minimum ratio count of 3. The first search peptide tolerance was set to 20, the main search peptide tolerance to 4.5 ppm, and the re-quantify option was selected. For peptide and protein identification, the human subset of the SwissProt database (release 2019_11) that included HPV protein sequences was used and contaminants were detected using the MaxQuant contaminant search. A minimum peptide number of 2 and a minimum length of 7 amino acids were tolerated. Unique and razor peptides were used for quantification. The match between run option was enabled with a match time window of 0.7 min and an alignment time window of 20 min. The statistical analysis including ratio and significance. A calculation was done using the Perseus software (version 1.6.2.3 [66]).

### Strand-specific transcriptomic data analysis

Total RNA was isolated from HPV16 positive keratinocytes using the RNeasy minikit (Qiagen). High RNA integrity was confirmed by applying a TapeStation RNA Assay (Agilent, 5067-5577-5581, sample RIN 7.5-9.0). Per sample, 90–520 ng total RNA (quantified by Qubit RNA HS Assay, Thermo Fisher, Q32852) was rRNA depleted applying the NEBNext rRNA Depletion Kit Human/Mouse/Rat (NEB, product number E6310L) and further processed via the NEBNext Ultra II Directional RNA Library Prep Kit for Illumina (NEB, catalog number E7760L) according to manufacturer’s instructions (with 8 min fragmentation time and applying 9 or 13 cycles of PCR enrichment of adaptor ligated DNA, step 4.8, instruction manual version 6.0 7/24).

Libraries were quality controlled on a TapeStation D1000 Assay (Agilent, D1000 ScreenTape 5067-5582 with D1000 Reagents 5067-5583) and were sequenced on an Illumina NextSeq2000 with the NextSeq 2000 P3 Reagents (100 Cycles) kit (product number: 20040559) in a paired-end mode (2 × 57 bp). After demultiplexing via bcl2fastq, 45.7–61.2 million read pairs assigned to each sample. All samples passed quality control by fastqc and were subjected to downstream analysis.

Strand-specific gene counts/abundance for each condition and replicate were quantified using STAR (67) and a custom reference of human GRCh38 genome assembly and HPV16 genbank reference K02718.1 in combination with the human gene annotation from ENSEMBL/GENCODE (version 105) and viral annotation. Human count normalization and differential gene expression analysis were performed using DESeq2 package (68). FDR (BH-adjusted *P*-values) <0.1 and log2foldchange < −1 and >1 was used as criteria for the final DE gene list. Gene ontology enrichment analysis was performed on the obtained gene list using topGO (69). GO plots for enrichment of biological process were plotted using R package clusterProfiler (70).

Quantification of HPV16 ORF sense and antisense transcription was performed using the STAR quantification results for sense and antisense fragments generated in the steps described above.

## Data Availability

Proteomics data have been deposited in the MassIVE (https://massive.ucsd.edu/) database under the accession number MSV000098431. Next generation sequencing sets have been deposited in the ENA (https://www.ebi.ac.uk/ena/) database under the accession number PRJEB91709.
